# Two-year incidence and risk factors of diabetic foot ulcer: second phase report of Ahvaz diabetic foot cohort (ADFC) study

**DOI:** 10.1186/s12902-024-01572-x

**Published:** 2024-04-15

**Authors:** Leila Yazdanpanah, Hajieh Shahbazian, Saeed Hesam, Behnam Ahmadi, Amir Mohammad Zamani

**Affiliations:** 1 Diabetes Research Center, Health Research Institute, Ahvaz Jundishapur University of Medical Sciences, 61357-15794 Ahvaz, Iran; 2 Department of Epidemiology and Biostatistics, School of Public Health, Tehran University of Medical Sciences, Tehran, Iran; 3 Student Research Committee, Ahvaz Jundishapur University of Medical Sciences, Ahvaz, Iran

**Keywords:** Incidence, Risk factors, Diabetic foot ulcer, DFU

## Abstract

**Aim/Introduction:**

This study was designed as the second phase of a prospective cohort study to evaluate the incidence and risk factors of diabetic foot ulcers (DFU).

**Materials and methods:**

The study was conducted in a university hospital in Iran. Each participant was checked and followed up for two years in terms of developing newfound DFU as ultimate outcome. We investigated the variables using univariate analysis and then by backward elimination multiple logistic regression.

**Results:**

We followed up 901 eligible patients with diabetes for two years. The mean age of the participants was 53.24 ± 11.46 years, and 58.53% of them were female. The two-year cumulative incidence of diabetic foot ulcer was 8% (95% CI 0.071, 0.089) [Incidence rate: 49.9 /1000 person-years]. However, the second-year incidence which was coincident with the COVID-19 pandemic was higher than the first-year incidence (4.18% and 1.8%, respectively). Based on our analysis, the following variables were the main risk factors for DFU incidence: former history of DFU or amputation [OR = 76.5, 95% CI(33.45,174.97), *P* value **<** 0.001], ill-fitting foot-wear [OR = 10.38, 95% CI(4.47,24.12), *P* value **<** 0.001], smoking [OR = 3.87,95%CI(1.28, 11.71),*P* value = 0.016], lack of preventive foot care [OR = 2.91%CI(1.02,8.29),*P* value = 0.045], and insufficient physical activity[OR = 2.25,95% CI(0.95,5.35),*P* value = 0.066].

**Conclusion:**

Overall, the two-year cumulative incidence of diabetic foot ulcer was 8% [Incidence rate: 49.9 /1000 person-years]; however, the second-year incidence was higher than the first-year incidence which was coincident with the COVID-19 pandemic (4.18% and 1.8%, respectively). Independent risk factors of DFU occurrence were prior history of DFU or amputation, ill-fitting footwear, smoking, lack of preventive foot care, and insufficient physical activity.

## Introduction

Diabetes continues to be a significant public health problem. There are some diabetes-related complications which increase with the rising prevalence of diabetes all over the world [1, 2]. Diabetes has been identified as the most common fundamental factor accounting for lower-extremity amputation in the U.S. and Europe [2, 3]. Many risk factors including peripheral ischemia, neuropathy, foot deformity, trigger diabetic foot ulcer (DFU). Ulcer healing is a time-consuming process and may even result in amputations so much so that one-third of ulcers never repair [4, 5]. In the meantime, up to one-fourth of diabetic patients are likely to develop DFU [2, 6–8]. Every 20 s, a diabetes-related amputation is done around the globe [9, 10]. Diabetes amputations raise the mortality rate. The 5-year survival in patients experiencing DFU is 70%, but following a major amputation, it deteriorates to 43%. The 5-year mortality in individuals with DFU is 2.5 times higher than that in diabetic patients without DFU [10–12]. The economic burden of DFU imposed on health care systems, including direct and indirect costs, is alarmingly huge. DFU significantly contributes to the worldwide burden of disability and diminishes the quality of life.

Diabetic foot ulcer treatment is time-consuming and demanding. Fortunately, however, the occurrence of foot ulcers is preventable. Prior studies have stated that early detection of patients at risk of foot ulcers and management of risk factors could avoid amputations and foot ulcerations. Therefore, prediction of risk factors in patients with DFU facilitates disease management for clinicians to select the best strategy [4]. Unfortunately, there are few significant cohort studies on DFU incidence [13–17]. In our country, Iran, no cohort study has been conducted on DFU incidence and risk factors. Additionally, socio-economic differences among different populations may have an effect on the incidence rates. The Ahvaz Diabetic Foot Cohort (ADFC) study was the first prospective study in Iran to evaluate DFU incidence and its risk factors [18]. The present study reports the results of the second phase of this investigation to help policy-makers in the region make practical and effective decisions based on the obtained outcomes.

### Subjects

The first phase of ADFC which is a population-based prospective cohort study was conducted between 2014 and 2016, and its results have already been published [18].

**Fig. 1:**
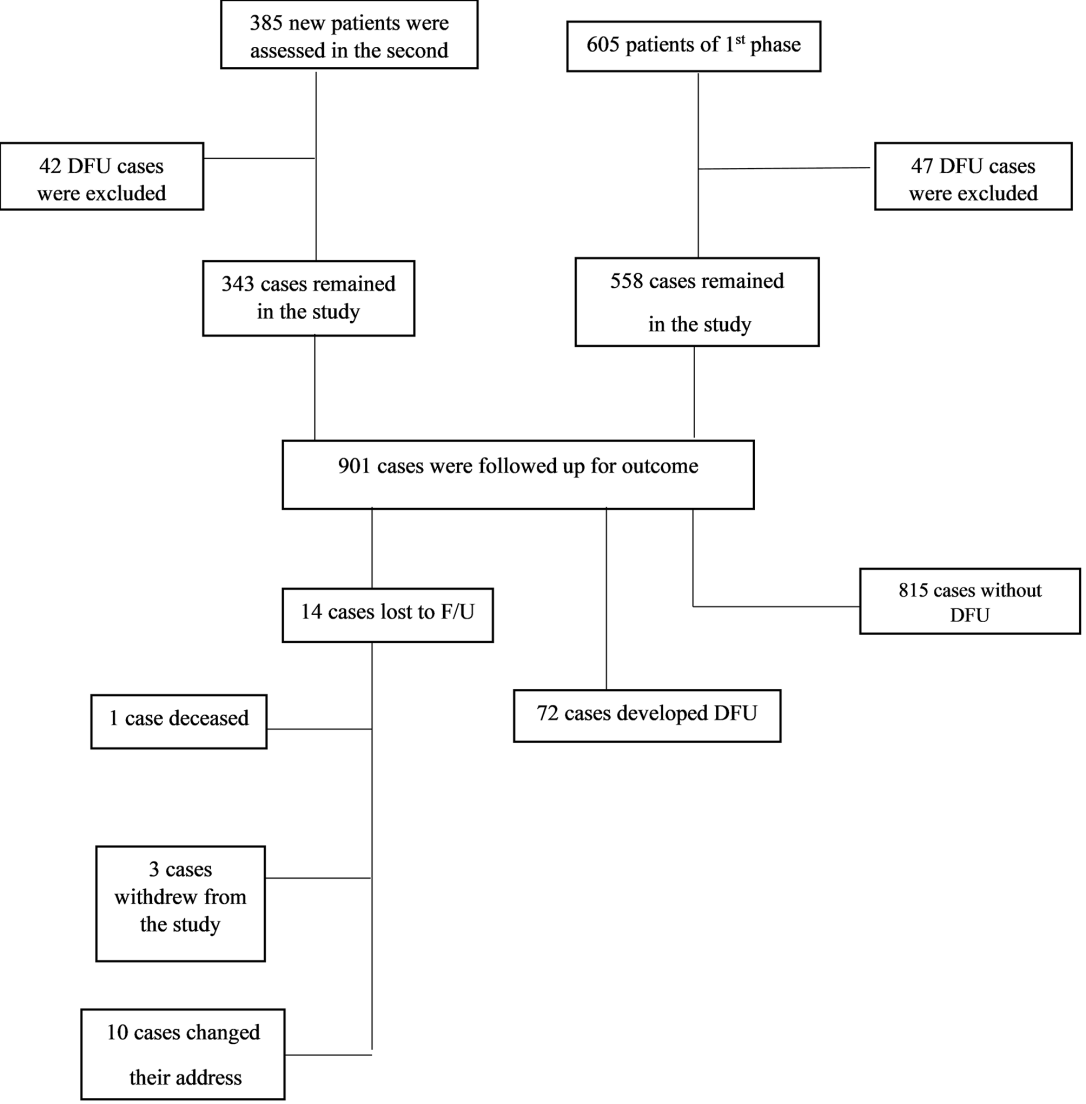
Diagram of the participants of the second phase of ADFC

The second phase of this study was conducted on the same population i.e., patients referring to the Diabetic Foot Clinic in Golestan Hospital, a university hospital in Ahvaz, southwest of Iran, from October 2019 to October 2021. We evaluated all diabetic patients referring to this clinic, which is the first diabetic foot clinic in the region. Of the 605 patients assessed in the first phase, 47 who had DFU at the beginning of the study in October 2019 were excluded. We assessed other 385 new cases in the second phase of whom 28 DFU cases were excluded. Finally, we followed up 901 cases for the outcome (i.e., new diabetic foot ulcer) (Fig. 1).

## Materials and methods

Non-probabilistic convenience sampling was used to select the patients. The inclusion and exclusion criteria of the study remained the same for the second phase without any changes [18]. Participants had to meet the following inclusion criteria: (1) be 18 years or older, (2) have been diagnosed with diabetes mellitus (both type 1 and 2), (3) be able to complete a consent form, and (4) be able to walk. Exclusion criteria included (1) having a severe disabling disease or inability to walk, (2) having a severe mental illness that would prevent informed consent, and (3) currently having a foot ulcer. The research was approved by the Ethics Committee of Ahvaz Jundishapur University of Medical Sciences. The method of the study was explained to all patients who later signed a written informed consent form. We prepared a checklist which was completed by all participants. It included information about sex, age, blood pressure (BP), educational attainment, marital status, ethnicity, body mass index (BMI), smoking status, type of diabetes, diabetes duration, diabetes treatment type (oral anti-diabetes agents or insulin consumption), diabetic nephropathy, diabetic retinopathy, history of DFU or amputation, availability of preventive foot care, patient training about their feet, nail care, and ill-fitting shoes.

Educational attainment was classified as: illiterate, high school diploma, and university degree. Blood pressure was recorded as systolic and diastolic BP using a mercury sphygmomanometer. Marital status was defined as: single, married, widow (or widower), or divorced. Ethnicity was categorized as: Fars, Arab, Lor, and Other. BMI was measured in kg/m2. Smoking status was expressed as: present smoker, ex-smoker, and no history of smoking. Diabetic retinopathy was taken into account if the patients’ medical documentation included pupil dilation followed by evaluation by fundoscopy (non-proliferative or proliferative retinopathy, clinically significant macular edema). Diabetic nephropathy was described based on 24-hour urine collection test with microalbuminuria or overt proteinuria and/or azotemia, dialysis, or kidney transplantation. Exercise was defined as regular physical activity of at least 30 min every other day. Preventive foot care involved: washing the feet and looking after the feet every day, wiping the feet after washing, moisturizing the feet, not walking barefoot, not putting the feet close to the heater, and wearing slippers and appropriate socks at home. If patients performed four or more of these actions, we considered their preventive foot care as sufficient. Performing fewer than four of these activities was regarded as insufficient foot care. Nail care was described as: not to cut toenails too short or trim the corners of the toenails. Fit footwear refers to the sufficiency of foot-wear length, width, and height according to foot size [6]. Ill-fitting footwear in this study was defined as: slippers, tight shoes, or shoes with forced points on the feet. Proper socks were considered as cotton socks having a flexible elastic band. Patient training on feet was described as: self-educating (reading books or pamphlets, visiting websites, or watching videos) or attending programmed individual or group classes.

After completing the checklist, all participants were examined by trained general physicians. The examination included: skin and nails, foot deformity type, neurologic foot assessments, and vascular foot tests. DFU was considered as a full-thickness skin defect receiving at least a Wagner gradeof 1 [19]. Protective sensation was examined by 10-gram monofilaments (Owen Mumford, UK). Nylon monofilaments were used vertically on four sites (1st, 3rd and 5th metatarsal heads and plantar surface of distal hallux) of each foot. We did not place the monofilament on ulcers, calluses, necrotic tissues or scars. Distal neuropathy was confirmed if the patient could not detect even one position of examination by monofilament [20]. We assessed dorsalis pedis, tibialis posterior, popliteal and femoral pulses in vascular evaluation. ABI (Ankle-brachial index) was calculated using a handheld Doppler device (Hunt-Ligh Diabetic Foot Kit, UK) and based on the following formula: ABI = (highest systolic pressure of dorsalis pedis artery or tibialis posterior) / (highest systolic pressure of brachial artery) one by one for both legs. The normal range of ABI was considered 0.9–1.3, ABI = 0.4–0.9 (vascular disease) and ABI < 0.4 (severe vascular disease) [21].

The patients’ HbA1c level was recorded based on their last medical test. Glycemic control was satisfactory, moderately good, and poor if HbA1c was less than 7%, 7–8%, and more than 8%, respectively [22].

Finally, every patient was followed up for two years for a new DFU as the outcome.

The method of follow-up involved monthly phone calls, and patients with new ulcers were invited to come to the clinic for a new examination.

We used SPSS version 20 to analyze the data. Continuous variables were described as mean ± SD, and frequency and percentage were used to describe categorical data. The variables were initially calculated as univariate analysis. The statistical tests used for this purpose were independent t-test (Mann-Whitney test if the data were not normally distributed) and chi-square test. Variables were analyzed by multivariate analysis using backward elimination multiple logistic regression. The most statistically significant variables were recognized as risk factors. *P* value ≤ 0.05 was considered statistically significant.

## Results

### Clinical characteristics of all participants

Of all 990 participants enrolled in this study, 901 were eligible and were followed up for two years (Fig. 1). The mean (± SD) age of the participants was 53.24 ± 11.46 years. Of all cases, 525(58.53%) were female. The mean (± SD) duration of diabetes was 9.4 ± 6.8 years, and the mean HbA1c was 8.63 ± 1.75%, with more than half of the patients having poor glycemic control (553 cases (61.4%)).

All patients were followed up to check the development of diabetic foot ulceration as an outcome. The two-year cumulative incidence of DFU was 8% (95% CI 0.071, 0.089) (72 cases). [Incidence rate: 49.9 /1000 person-years]. The one-year incidence (risk) of diabetic foot ulcer was 1.8% (95% CI 0.009, 0.027) (16 cases) whereas the second-year incidence was 4.18% (95% CI 0.029, 0.055).

### Comparison of clinical characteristics in groups with and without DFU

We excluded from data analysis patients who were lost to follow-up (14 cases). The baseline characteristics of all participants and a comparison between the two groups (with and without diabetic foot ulcers) are shown in Tables 1 and 2. The univariate analysis demonstrated that the following variables are significantly related to DFU development: male gender, diabetic neuropathy, history of DFU or amputation, foot care education, ill-fitting footwear, and exercise (Table 1).

**Table 1:** Baseline characteristics of all participants and comparison between the two groups (developing and not developing DFU) based on quantitative variables

Characteristics	All patients (*n* = 901)	Patients developing DFU (*n* = 72)	Patients Not developing DFU (*n* = 829)	OR (95%CI)	*P* value
*Age(Year)*	53.24 ± 11.46	53.94 ± 12.92	53.18 ± 11.33	1.01(0.99,1.03)	0.586
*Diabetes duration(Year)*	9.4 ± 6.8	9.51 ± 6	9.38 ± 7.0	1(0.997,1.003)	0.873
*BMI(kg/m* ^*2*^ *)*	28.59 ± 4.66	28.34 ± 5.55	28.61 ± 4.58	0.99(0.94,1.04)	0.632
*Blood pressure(mmhg)*					
Systolic BP Diastolic BP	127.84 ± 13.55 80.09 ± 6.24	127.99 ± 15.49 80.28 ± 6.65	127.82 ± 13.37 80.08 ± 6.20	1.001(0.98,1.0) 1.005(0.97,1.0)	0.922 0.801
*HbA1c (%)*	8.63 ± 1.75	8.84 ± 1.97	8.61 ± 1.73	1.08(0.94,1.23)	0.291

DFU: diabetic foot ulcer, BP: blood pressure (variables are expressed as mean ± SD)

Univariate evaluation of risk factors of the incidence of DFU and the comparison between patients with and without DFU is presented in Tables 1 and 2.

**Table 2:** Univariable assessment of risk factors of diabetic foot ulcer incidence and comparison between patients with and without DFU based on qualitative variables

Variable*	All patients (*n* = 901)	Patients with DFU (*n* = 72)	Patients without DFU (*n* = 829)	Unadjusted OR (95%CI)	*P* value
*Gender*					
Female Male	525(58.3) 376(41.7)	38(7.2) 34(9)	487(92.8) 342(91)	Reference 1.27(0.79,2.07)	0.326
*Education*					
Illiterate ≤High school Diploma University degree	229(25.4) 595(66) 77(8.5)	24(10.5) 39(6.6) 9(11.7)	205(89.5) 556(93.4) 68(88.3)	0.89(0.39,1.99) 053(0.25,1.14) Reference	0.768 0.105
*Ethnicity*					
Fars Arab Lor other	241(26.7) 500(55.5) 137(15.2) 23(2.6)	18(7.5) 45(9) 8(5.8) 1(4.3)	223(92.5) 455(91) 129(94.2) 22(95.7)	1.3(0.55,3.08) 1.59(0.73,3.47) Reference 0.73(0.09,6.15)	0.548 0.239 0.775
*Marital status*					
Single Married Divorced or widow	26(2.9) 539(93.1) 36(4)	2(7.7) 67(8) 3(8.3)	24(92.3) 772(92) 33(91.7)	Reference 1.04(0.24,4.5) 1.09(0.17,7.04)	0.957 0.927
*Smoking status*					
Smoker Ex-smoker Non-smoker	44(4.9) 66(7.3) 791(87.8)	6(13.6) 7(10.6) 59(7.5)	38(86.4) 59(89.4) 732(92.5)	1.96(0.79,4.82) 1.47(0.64,3.37) Reference	0.144 0.360
*Neuropathy*					
Yes No	332(36.8) 569(63.2)	35(10.5) 37(6.5)	297(89.5) 532(93.5)	1.69(1.05,2.75) Reference	**0.033**
*Nephropathy*					
Yes No	56(6.2) 845(93.8)	6(10.7) 66(7.8)	50(89.3) 779(92.2)	1.42(0.59,3.43) Reference	0.440
*Retinopathy*					
Yes No	204(22.6) 697(77.4)	18(8.8) 54(7.7)	186(91.2) 643(92.3)	1.15(0.66,2.01) Reference	0.618
*History of previous DFU or Amputation*					
Yes No	55(6.1) 846(93.9)	41(74.5) 31(3.7)	14(25.5) 815(96.3)	76.99(38.05,155.79) Reference	**< 0.001**
*Oral glycemic agents consumption*					
Yes No	662(73.5) 239(26.5)	51(7.7) 21(8.8)	611(92.3) 218(91.2)	Reference 1.15(0.68,1.96)	0.597
*Insulin consumption*					
Yes No	260(28.9) 641(71.1)	27(10.4) 45(7)	233(89.5) 596(93)	1.54(0.93,2.53) Reference	0.093
*Foot Deformity*					
Yes No	93(10.3) 808(89.7)	11(11.8) 61(7.5)	82(88.2) 747(92.5)	1.64(0.83,3.25) Reference	0.153
*ABI*					
Normal Abnormal	884(98.1) 17(1.9)	69(7.8) 3(17.6)	815(92.2) 14(82.4)	Reference 2.53(0.71,9.02)	0.152
*Exercise*					
Yes No	271(30.1) 630(69.9)	12(4.4) 60(9.5)	259(95.6) 570(90.5)	Reference 2.27(1.2,4.3)	**0.012**
*Preventive foot care*					
Yes No	111(12.3) 790(87.7)	13(11.7) 59(7.5)	98(88.3) 731(92.5)	1.64(0.87,3.11) Reference	0.126
*Patient education on foot care*					
Yes No	312(34.6) 589(65.4)	14(4.5) 58(9.8)	298(95.5) 531(90.2)	Reference 2.33(1.28,4.24)	**0.006**
*Ill-fitting footwear*					
Yes No	360(40) 541(60)	59(16.4) 13(2.4)	301(83.6) 528(97.6)	7.29(4.29,14.75) Reference	**< 0.001**

* Variables are reported as N (%)

### Analysis of the risk factors of DFU using multivariate logistic regression

For multivariate logistic regression analysis, we used backward elimination method. Table 3 shows the risk factors that remained in the regression model. In summary, history of previous DFU or amputation, ill-fitting footwear, smoking, loss of preventive foot care, and decreased physical activity had a statistically significant relationship with DFU incidence.

After adjustment for other variables, former history of DFU or amputation led to a 76.5-fold increase in the odds of DFU in comparison with patients who did not experience DFU (the highest risk). The odds of DFU in cases using unsuitable shoes were 10.38 times greater than those in patients using well fitted foot-wear. The odds of DFU were 3.87 times more in smokers compared with non-smokers. DFU was 2.91 times more expected in patients who had inadequate preventive foot care compared with patients who committed themselves to having proper and sufficient personal foot care. Patients with irregular and insufficient physical activity were 2.25 times more likely to develop DFU in comparison with patients with adequate exercise (Table 3).

**Table 3:** Independent risk factors of DFU (multivariable logistic regression analysis)

Risk factors	Adjusted OR	95%CI	*P* value
*History of previous DFU or Amputation*			
Yes No	76.5 Reference	(33.45,174.97)	< 0.001
*Ill-fitting footwear*			
Yes No	10.38 Reference	(4.47,24.12)	< 0.001
*Preventive foot care*			
Yes No	Reference 2.91	(1.02,8.29)	0.045
*Exercise*			
Yes No	Reference 2.25	(0.95,5.35)	0.066
*Smoking status*			
Smoker Cessation Non-smoker	3.87 0.58 Reference	(1.28,11.71) (0.15,2.23)	0.016 0.428

OR: Odds Ratio, CI: Confidence Interval

A comparison between variables of the first and second phases of the ADFC study showed no significant differences between participants of the two phases in terms of their age and diabetes duration (*P* = 0.643 and 0.098, respectively). A comparison between qualitative variables of the two phases of the study is shown in Table 4.

**Table 4:** Comparison of ADFC study’s first and second phase in terms of qualitative variables

Variable	First Phase N (%)	Second Phase N (%)	*P* value
Diabetic Neuropathy	172(32.2)	332(36.8)	0.075
Diabetic Retinopathy	106(19.9)	204(22.6)	0.214
Diabetic Nephropathy	47(8.8)	56(6.2)	0.067
Abnormal ABI	6(1.1)	17(1.9)	0.266
Foot Deformity	50(9.4)	93(10.4)	0.558
Positive History of previous DFU or Amputation	11(2.1)	55(6.1)	**< 0.001**
Insulin consumer	163(30.5)	260(28.9)	0.503
Smoker	26(4.9)	44(4.9)	0.881
Trained about foot care	77(14.4)	312(34.6)	**< 0.001**
Ill-fitting footwear	338(63.3)	360(40)	**< 0.001**
Having preventive foot care	4(0.7)	111(12.3)	**< 0.001**

## Discussion

ADFC (Ahvaz Diabetic Foot Cohort) study is the first study to evaluate diabetic foot incidence in the southwest of Iran [18]. In the present study, which is the second phase of ADFC, we aimed to investigate the incidence and risk factors of DFU in a two-year follow-up. As our findings showed, the two-year cumulative incidence of DFU was 8% in this region (95% CI 0.071, 0.089) [Incidence rate: 49.9 /1000 person-years].

Expected risk factors were evaluated among patients who had developed DFU and those who had not. After multivariate analysis, independent risk factors of DFU in this study were found to be: a history of earlier DFU or amputation, ill-fitting footwear, smoking, loss of preventive foot care, and decreased physical activity.

In the first year of follow-up, DFU incidence was 1.8% (95% CI 0.009, 0.027) which was lower in comparison to ADFC’s first phase incidence [5.62(95% CI3.89, 8.02)] [18]. This reduction can be due to the establishment of a separate unit serving as a diabetic foot clinic in the Golestan Diabetes Clinic for the first time. This unit focused on educating patients on their feet, which had long been a neglected issue in diabetes management in the region.Therefore, the decrease in the incidence of DFU could be attributed to this education which involved topics such as preventive foot care, self-management, and glycemic control besides holding workshops for physicians and nurses. However, the overall incidence was higher compared with the first year. It is important to note that the second year of follow-up was coincident with the COVID-19 pandemic, which may be a probable reason for a higher overall DFU incidence in the second phase of ADFC in comparison with the first phase (8% and 5.62% respectively). The rate of on-time appointments in clinics decreased during the pandemic, and this may have contributed to inadequate education and unsuitable glycemic levels. Moreover, prolonged quarantines during the pandemic period limited the patients’ physical activity, which could have deteriorated their glycemic control and enhanced the risk of complications in some way.

Prospective studies on DFU incidence are scarce [13–17]. The overall DFU incidence in our study was close to the results of some previous studies [23]. Another investigation reported different incidence rates. Studies in Japan (incidence rate:2.9/1000 person-years) [24], the UK (1.93% annual incidence) [25], the UK (2.2% average annual incidence) [26], and Ethiopia ( incidence rate of 4 cases per 100 person-years of observation) [27] reported incidences lower than ours, while the following studies found higher incidences: the US (5.8%) [28], China (8.1% annual incidence) [29], UK (incidence rate 11.1 to 6.1 per 1000 persons between 2003 and 2017) [17].

Many studies have evaluated the effect of the COVID-19 pandemic on glycemic control in patients with diabetes. However, there are few studies addressing the impact of this pandemic on diabetes complications. One study conducted in Indonesia in 2021 demonstrated that diabetes complications were 1.41 times higher during the pandemic based on multivariate analysis [95%CI: 1.09–1.83] [30]. Liu’s study reported the significant effect of the COVID-19 pandemic on DFU development [31]. Some other studies reported higher DFU emergencies, amputation and mortality in comparison to before and after the lockdown [32–34]. The first reports of elevated rates of amputations during the pandemic were from Italy and the U.S [33]. Induced changes in patient management such as online visits were hardly welcomed, especially by older patients who were not familiar enough with novel technologies. All of these led to a decrease in the care of diabetic patients, which may explain the explosive increase in DFU incidence during the pandemic in the present study. In contrast with several previous studies, however, a few studies, such as Falcetta’s study in Italy, reported no destructive consequence of lockdown on glycemic control in patients with diabetes [35].

Different risk factors contributing to DFU development have been reported in separate studies [36]. However, some factors are more frequently cited in different studies. We analyzed the data in univariable and multivariable logistic regression models. The final DFU risk factors in this study were: a history of previous DFU or amputation, ill-fitting footwear, smoking, loss of preventive foot care, and decreased physical activity.

The most correlated risk factor of DFU in the present study was former history of DFU or amputation, which was consistent with many studies [2, 3, 24, 37].

In this study, the mentioned risk factor increased the odds of ulceration 76.5 times higher in comparison to patients without DFU history. Moreover, according to the results of the first phase of ADFC, the odds of DFU development are 25 times higher in patients with such a history. Patients with a history of DFU or amputation may be prone to several micro- and macro-vascular complications such as diabetic neuropathy and ischemia, which may explain the higher threat of subsequent ulcers. In a meta-analysis conducted in 2018, previous history of DFU had the highest odds of ulcer development among all other independent risk factors [OR = 6.59(95% CI: 2. 49, 17. 45)] [38]. Nevertheless, in a prospective cohort study in Tanzania, history of DFU or amputation was significantly related to ulcer development only based on univariable analysis, but it did not remain in the model after multivariable logistic regression [39].

Properly fitting footwear is an essential element in preventing DFU by lowering inflammation and callus formation. The exact number of ulcers initiated with unsuitable footwear (material or condition) is unknown. However, according a previous study, 40% of ulcers appeared at the hallux, 13% on the dorsum of digits, and 10% on the plantar side of digits. These are possible sites that may be affected by footwear abrasion, which will lead to ulcer development. External traumas are described as factors frequently contributing to DFU. Minor trauma can be ill-fitting footwear wherein the soft tissues of the foot remain weight-bearing for a long time [6, 40, 41]. Ill-fitting footwear increased the odds of foot ulcer occurrence by more than 10 times in this study even though it was not significantly related to DFU in the first phase of ADFC (*P* = 0.433). A comparison between the two phases of ADFC shows that inappropriate footwear decreased significantly (*P* < 0.001) as shown in Table 4. This is despite the fact that wearing slippers is common due to cultural issues and because of the unbearably hot weather in the southwest of Iran. Overall, some more controllable factors such as patient training, ill-fitting footwear, and preventive foot care, which are highly sensitive to education, have a more acceptable condition in the second phase of ADFC (Table 4).

After adjustment for other variables in this study, smokers had 3.87 times higher odds of developing DFU in comparison to non-smokers. This gives the impression that there may be an association between smoking and the male gender in the assessed population since male gender was correlated significantly with DFU in multivariable analysis in the first phase of ADFC, and smoking was excluded after backward elimination Additionally, in this phase, smoking was superior to gender, and only smoking remained in the model while gender was excluded. It should be noted that in the Iranian culture, most smokers are men, which may clarify why only one of these two variables (smoking and male gender) remained in the regression model ultimately. In a retrospective study in Albania in 2021, smoking remained significant in multivariable analysis, which is consistent with our results [42]. Furthermore, in Alberta’s Caring for Diabetes (ABCD) study, smoking was reported as a predictive factor for DFU development [37]. A systematic review and meta-analysis suggested that smoking had a damaging influence on the healing of DFU [43]. On the contrary, in Tanzania prospective cohort study, smoking was a predisposing factor of DFU only in univariable analysis but not in the multivariate investigation [39]. In Bin Hameed’s study, smoking had no significant contribution to DFU occurrence [44].

In this study, in patients having insufficient preventive care of their feet, the odds of DFU occurrence were 2.91 times more compared with patients with sufficient foot care. According to Table 4, patients had significantly less preventive foot care in the first phase compared with the second phase (*P* < 0.001). As mentioned earlier, this may be a long-lasting outcome of education in the diabetic foot clinic. The influence of foot care education on preventive foot care has already been confirmed in patients with diabetes [45–47]. Foot care education had a statistically significant relationship with DFU episodes in multivariate analysis in the first phase of the study, but in the second phase, loss of preventive foot care remained significant in the regression model instead. There is probably a relationship between these two variables (patient education and preventive foot care), which is logical. Consistent with our results, a meta-analysis in the Ethiopian population conducted in 2020 demonstrated that the presence of callus on feet (as a result of loss of preventive foot care) and poor self-care practice increased the odds of DFU development [ (OR = 12.67,95% CI:6.47–24.70 ) and (OR = 1.47, 95% CI:1.25–1.73), respectively] [48]. In addition, Naemi et al. found that the presence of callus was statistically associated with DFU based on the multivariable investigation [39]. In the ABCD study [37], the authors found that patients with low self-efficacy are nearly twice as expected to develop foot problems than those having high self-efficacy. The variable of self-efficacy in their research was partly similar to the variable of preventive foot care in our study. In the second phase of ADFC, the number of patients who had preventive foot care was higher compared with the first phase, so their evaluation had more precision. Variable unbalance was higher in the first phase due to the small sample size, which may result in finding no relationship between DFU and preventive foot care. In other words, the correlation between DFU and preventive foot care in the second phase was more accurate than the absence of the relationship between these two variables in the first phase.

The participants with irregular and insufficient physical activity were 2.25 times more likely to develop DFU than were patients with regular exercise in their lifestyle. Of course, we did not assess this variable in the first phase, but based on our experience in the two phases of the study and reviewing other studies, it seems that this variable may contribute to DFU due to its direct and indirect consequences on blood glucose levels and glycemic control. Results of a systematic review assessing only controlled clinical trials showed that physical activity can be effective in the outcome of DFU and its incidence [49]. Indeed, exercise can improve not only nerve velocity conduction, peripheral sensory function, and foot peak pressure but also Ankle Brachial Index (ABI) [50]. Physical activity could have remained significant in the logistic regression model, had these effects been taken into account. Undoubtedly, no evidence-based recommendation has so far been put forward regarding the benefit or harm of physical activity after DFU occurrence [51].

This study has some limitations. Firstly, selecting participants from a university hospital may disturb the outcomes due to selection bias. Of course, the hospital from which the data of the present study was collected was the referral focal point of diabetes in the province which can be considered the strength of the study, but this can simultaneously reduce generalizability of the results. The next limitation was self-reported data for some variables like preventive foot care which was subject to recall bias. Besides, we did not take into account some probable confounders in DFU development such as patient communicative factors like compliance with education about their foot care.

What makes this study particularly worthwhile was its larger sample size in the first phase and the low rate of loss to follow-up in comparison to other studies [37–43]. This study was the first population-based prospective cohort of diabetic foot in Iran with participants who were followed up for about seven years from the first phase of the study. We tried to control some limitations of the first phase and add some other variables like physical activity. These results may provide new information on the predictors of DFU and implications for future research. Moreover, such information could provide practical evidence to help policy-makers in the region arrange effective decisions based on the obtained outcomes.

Early purposive screening according to predicted factors might detect patients needing additional support in the follow-up care. Consequently, these activities may reduce DFU incidence and its huge economic burden in the region.

We recommend further studies with a larger sample size in future. According to the high DFU incidence after the pandemic in this study, we recommend evaluating the role of telemedicine in this topic in future studies.

## Conclusion

To sum up, the cumulative two-year incidence of diabetic foot ulcer was 8% [Incidence rate: 49.9 /1000 person-years] while the second-year incidence was higher than that of the first year which was coincident with the COVID-19 pandemic (4.18% and 1.8% respectively). Independent risk factors of DFU occurrence were prior history of previous DFU or amputation, ill-fitting footwear, smoking, loss of preventive foot care, and decreased physical activity.

## Data Availability

The datasets used and/or analyzed during the current study are available from the corresponding author on reasonable request.

## Abbreviations

DFU: Diabetic foot ulcer
ADFC: Ahvaz diabetic foot cohort
CI: Confidence interval
SD: Standard deviation
BP: Blood pressure
ABI: Ankle brachial index
OR: Odds ratio
N: Number
